# Potassium stress growth characteristics and energetics in the haloarchaeon *Haloarcula marismortui*

**DOI:** 10.1007/s00792-014-0716-z

**Published:** 2014-12-11

**Authors:** Matthew W. Jensen, Scott A. Matlock, Carlene H. Reinheimer, Caleb J. Lawlor, Travis A. Reinheimer, Andrea Gorrell

**Affiliations:** Department of Chemistry, University of Northern British Columbia, 3333 University Way, Prince George, BC V2N 4Z9 Canada

**Keywords:** Haloarchaea, *Haloarcula marismortui*, Potassium, Growth, Ion transport, Potassium stress

## Abstract

Growth characteristics surrounding halophilic archaeal organisms are extremely limited in the scientific literature, with studies tending toward observing changes in cellular generation times under growth conditions limited to changes in temperature and sodium chloride concentrations. Currently, knowledge of the ionic stress experienced by haloarchaeal species through an excess or depletion of other required ions is lacking at best. The halophilic archaeon, *Haloarcula marismortui*, was analyzed under extreme ionic stress conditions with a specific focus on induced potassium ion stress using growth curves and analysis of the intracellular ion concentrations. Generation times were determined under potassium chloride concentrations ranging from 8 to 720 mM, and also in the presence of the alternative monovalent cations of lithium, rubidium, and cesium under limiting potassium conditions. Intracellular ion concentrations, as determined by inductively coupled mass spectrometry (ICP-MS), indicate a minimum intracellular total ion requirement of 1.13 M while tolerating up to 2.43 M intracellular concentrations. The presence of intracellular rubidium and cesium indicates that monovalent ion transport is important for energy production. Comparison of eight archaeal genomes indicates an increased diversity of potassium transport complex subunits in the halophilic organisms. Analysis of the generation times, intracellular concentrations and genome survey shows *Har. marismortui* exhibits an ability to cope with monovalent cation concentration changes in its native environment and provides insight into the organisms ion transport capability and specificity.

**Electronic supplementary material:**

The online version of this article (doi:10.1007/s00792-014-0716-z) contains supplementary material, which is available to authorized users.

## Introduction

Halophilic archaeal growth characteristics have not been well studied. One of the few investigations of haloarchaeal growth kinetics was conducted by Robinson et al. (2005), who found species of the family *Halobacteriaceae* possess cellular generation times varying from 1.5 to 3 h and several species within the family exhibit multiple temperature optima. The species most closely related to *Haloarcula marismortui* that they examined was *Haloarcula vallismortis*, which exhibits an optimal cellular generation time of 3.04 ± 0.20 h at an optimal temperature of 43–49 °C (Robinson et al. 2005). These past studies have examined growth characteristics dependent on temperature and sodium chloride concentration within the media; however, to the best of our knowledge, there has been no examination of changes in growth characteristics in response to ion stresses induced by specific ions normally present at lower concentrations in the growth media. The reported growth requirements of *Har. marismortui* are limited to an initial study by Ginzburg et al. (1970) stating a cellular generation time of 5–6 h for an unknown Dead Sea isolate and the proposal of this isolate as a novel species, *Har. marismortui*, in 1990 (Oren et al. 1990). This species is described as a highly pleomorphic cell type that demonstrates a tendency toward the rod shape and produces pink-colored cultures that deepen in color with age (Ginzburg et al. 1970). The past studies on *Har. marismortui* growth focused on substrate use and these publications are few (Brasen and Schonheit 2004; Ginzburg et al. 1970; Oren et al. 1990).

Ion transport in *Har. marismortui* has been studied to a far greater extent; Ginzburg et al. (1970) reported the first ion transport characteristics when a cell volume of 1.22 ± 0.02 mm^3^ (1.22 ± 0.02 μL) was calculated based on cell pellet density. They reported corresponding concentrations of potassium, sodium and chloride within *Har. marismortui*, using primarily gravimetric methodologies, of 3.7–5 M K^+^, 0.5–3 M Na^+^, and 2.3–4.2 M Cl^−^ (Ginzburg et al. 1970) across various stages of growth. Ginzburg et al. (1970) acknowledged that these concentrations were extreme and solutions of 4–5 M KCl and 1–3 M NaCl can not be prepared due to limitations of solubility, and further suggested that the potassium activity is limited within the cells, allowing for elevated concentrations. Moreover, the permeability of the membrane to larger biomolecules has lead to the suggestion that the mobility of potassium is largely restricted within the cells (Ginzburg 1969); however, no further studies have been conducted to confirm this suggestion to the best of our knowledge. This potassium restriction may be explained by a more recent neutron scattering study that examined cell water movement in *Har. marismortui* and revealed a slow-moving water component that accounts for approximately 76 % of the total cell water (Tehei et al. 2007). It has been suggested that this slow-moving water component is a solvation shell that is interacting with the large amounts of potassium bound to proteins (Tehei et al. 2007), due to the highly acidic proteome (Baliga et al. 2004).

As reviewed by Oren (1999), microorganisms in all domains of life utilize one of two primary mechanisms for survival in highly saline environments: (1) salt sequestration or (2) organic solutes. Cells can sequester salts internally to concentrations equivalent to, or higher than the extracellular salt concentrations forcing adaptation to a hyper-saline environment. Cells which balance osmotic pressure through organic solutes, such as glycerol, glycine betaine, or sucrose, eliminate the need for the adaptation of intracellular systems but require additional biosynthetic pathways. *Har. marismortui*, along with all other halophiles of the order *Halobacteriales,* use the first of these mechanisms for survival in their native hyper-saline environment (Oren 1999). Members of the order *Halobacteriales* utilize the proton electrochemical gradient across the cell membrane to drive the expulsion of sodium and sequestration of potassium (Mulkidjanian et al. 2008; Oren 1999; Schafer et al. 1999). This gradient is maintained via respiratory electron transport during aerobic growth or hydrolysis of ATP through membrane ATPases (Kakinuma and Harold 1985; Oren 1999). In the case of members of the family *Halobacteriaceae*, which includes *Har. marismortui*, the proton gradient can also be generated directly via the photosensitive proton pump, bacteriorhodopsin (Lanyi 2004; Oesterhelt and Stoeckenius 1971; Oren 1999). The established proton gradient, as maintained by any of the latter mechanisms, is then used in conjunction with Na^+^/H^+^ antiporters as a primary mechanism to drive and maintain the Na^+^ gradient across the cell membrane (Oren 1999). Additionally, the accumulation of Cl^−^ has been shown to occur via the photosensitive halorhodopsin transporter (Matsuno-Yagi and Mukohata 1980; Schobert and Lanyi 1982) and is also believed to occur through a co-transport mechanism with sodium to accomplish movement back into the cell (Oren 1999).

The accumulation of potassium has been argued to occur via passive diffusion through a uniport system allowing for accumulation proportional to the magnitude of the electrochemical potential across the cell membrane (Meury and Kohiyama 1989; Oren 1999). A study of potassium transport in the haloarchaeon, *Haloferax volcanii*, has shown, however, that the intracellular concentrations of potassium observed in this organism cannot be accounted for by passive processes alone and ATP hydrolysis is required to actively transport potassium into the cell to reach the 3.6 M intracellular concentrations that are maintained by *Hfx. volcanii* (Meury and Kohiyama 1989; Oren 1999). An ATP-regulated, low-to-medium affinity potassium transporter that is similar to the Trk system found in *Escherichia coli* has also been documented in halophilic species (Meury and Kohiyama 1989; Oren 1999) in addition to several other ion transporters/channels that contribute to overall potassium, sodium, and proton ion flow. The accumulation of intracellular potassium as a mechanism of osmoregulation is more energetically favorable than the mechanism of synthesizing or sequestering organic solutes [ATP:K^+^ costs reviewed by Oren (1999)]; however, adaptation of cellular processes to molar salt concentrations is required.

The primary aim of this study is to assess the cellular response to external potassium stress in *Har. marismortui*. Cellular generation times were evaluated across a variety of conditions that encompass extreme changes in extracellular potassium concentration and media pH. Growth in the presence of the alternative monovalent cations of lithium, rubidium, and cesium was also evaluated and the intracellular concentrations of potassium, lithium, rubidium and cesium ions were determined using inductively coupled plasma mass spectrometry (ICP-MS). Our results show *Har. marismortui* exhibits an ability to cope with monovalent cation concentration changes in its native environment and provides insight into the organisms ion transport capability and specificity.

## Materials and methods

### Materials

Chemicals were purchased from Sigma-Aldrich (Oakville, ON), unless otherwise noted.

*Haloarcula marismortui* (strain ATCC 4049) was obtained from Cederlane Biotechnologies (Cederlane, ON). Difco yeast extract, Difco agar and Oxoid peptone were purchased from Fischer Scientific (Ottawa, ON).

### Preparation of *Haloarcula marismortui* cell cultures

*Haloarcula marismortui* cells were grown in 23 % Salt Water Modified Growth Media (23 % S.W. MGM; 120 mM KCl) as previously described (Rodríguez-Valera et al. 1983, 1980) at 45 °C and 250 rpm (defined as standard growth conditions). The potassium contamination in standard purity NaCl is approximately 20 mM based on manufacturer’s batch analysis report. All KCl media constructed from these stocks includes this measurement, giving a final concentration of 120 mM KCl in standard media. Consistent lighting conditions were maintained throughout growth to eliminate the possibility of changes in cellular generation times due to changes in stimulation of photosensitive membrane proteins (bacteriorhodopsin (Oesterhelt and Stoeckenius 1971); halorhodopsin (Matsuno-Yagi and Mukohata 1980; Schobert and Lanyi 1982). Cells were continuously sub-cultured at mid-exponential growth (OD_600_ = 0.4–0.6) as a means of maintaining continuously doubling cultures. Once cultures had been sub-cultured no less than three times, cells were defined as being in “balanced growth”.

Media containing monovalent ions alternative to potassium was prepared as per the methods outlined previously (Rodríguez-Valera et al. 1983, 1980) with the following modifications: high-purity NaCl (99.9999 %, Fluka) was used in the preparation of the initial 30 % salt water solution, excluding any additional KCl and LiCl, RbCl or CsCl (3.5 M stock solution) was added to 120 mM final concentration. For LiCl media, the salt was added prior to autoclaving, while RbCl and CsCl, media was autoclaved prior to salt addition then sterile filtered. Due to trace quantities of potassium in the high-purity NaCl stock (99.999 %; 5 mg K/Kg NaCl) and concentrations of potassium in media components, we have conservatively estimated our maximum attainable potassium ion contamination in our media as 8 mM in lieu of costly trace metal batch analysis on high-sodium media. Media used for the assessment of growth at varying pH was prepared as per previously described methods (Rodríguez-Valera et al. 1983, 1980) then pH adjusted with HCl or Tris-base to a pH range from 2 to 7.5. Media above pH 7.5 were not assessed.

### Determination of cellular generation times

**Fig. 1:**
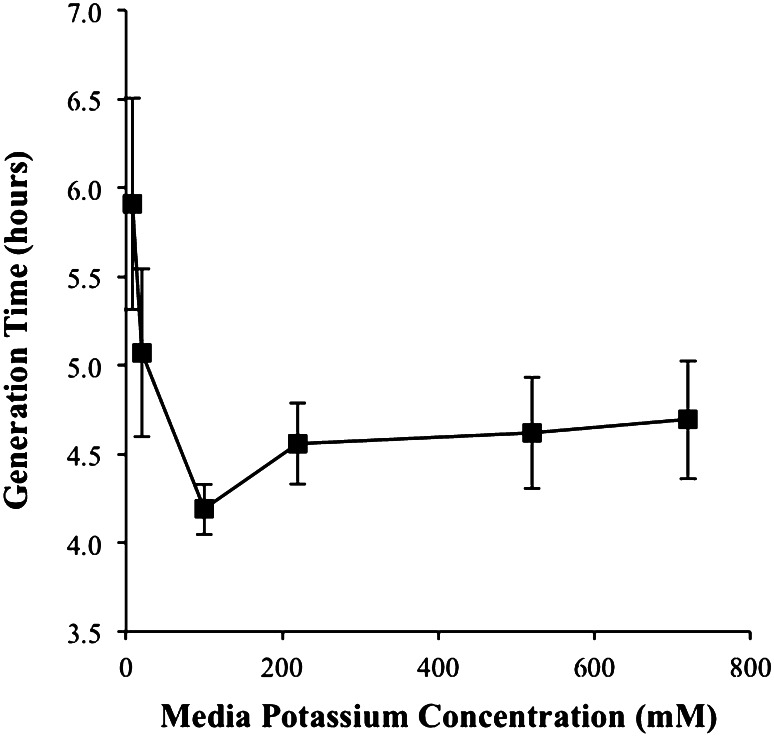
Generation times on different extracellular potassium concentrations. Values were obtained from the exponential growth curves via the exponential growth equation (Figure A2) vs. extracellular potassium concentration. *Error bars* represent standard error obtained from the determination of cellular generation times

Cells were grown to balanced growth in standard 23 % S.W. MGM as well as in media containing 8, 20, 220, 520, and 720 mM KCl. Each biological triplicate consisted of a technical triplicate and was monitored via spectrophotometry at 600 nm (OD_600_). Growth curves were constructed by measuring cell density at least once per generation time as estimated after a test culture. Measurements were averaged across all replicates and plotted against the growth time to determine the generation time (Fig. 1; Table 1).

**Table 1:** Cellular generation times observed under experimental growth conditions

Growth condition	Generation time (h)
8 mM KCl	5.91 ± 0.60
20 mM KCl	5.07 ± 0.47
120 mM KCl	4.19 ± 0.14
220 mM KCl	4.56 ± 0.23
520 mM KCl	4.67 ± 0.31
720 mM KCl	4.69 ± 0.33
120 mM LiCl*	16.90 ± 1.03
120 mM RbCl*	6.36 ± 0.13
120 mM CsCl*	23.23 ± 1.43

Values of for standard error were produced and obtained from the Kaleidograph software package. Growth conditions marked with asterik indicate a sudden stress condition in which cells were grown to balanced growth in standard 23 % SW. MGM before inoculation into test media

### Determination of cell density

The cell density of the pink, rod-shaped *Halobacterium* isolated from the Dead Sea has been previously reported as 1.20 g/mL by Ginzburg et al. (1970). As this isolate has been confirmed as being *Har. marismortui* by Oren et al. (1990), the techniques used in this report were modified and repeated to confirm this result. Cells (500 μL) at mid-exponential growth (OD_600_ = 0.4–0.6) and at saturation (OD_600_ > 0.7) were pelleted and resuspended in a small volume of 23 % S.W. MGM to create a high-viscosity cell suspension that was layered onto a range of sodium/sucrose solutions, with densities that were determined gravimetrically, and spun 30 s at 6000 rpm. The sucrose solution that, upon visual inspection, allowed 50 % of the suspension to travel 50 % of the height of sodium/sucrose solution was considered to be of equal density to the cells.

### Determination of average cell volume

Using the procedures for constructing a growth curve, optical densities were measured at least once every experimentally determined generation time. Cells were then diluted 50- to 1000-fold and counted using a standard hemocytometer. A standard curve plotting cell density against optical density of cell cultures grown under standard growth conditions was constructed and a line of best fit applied (Figure A1). The number of cells in a given volume of culture was determined using the straight line equation (*y* = 1.55 × 10^9 ^×  to −2.03 × 10^7^; *R* ^2^ = 0.9968) obtained from the standard curve. A volume of culture was centrifuged at 13,000 rpm for 5 min and the media was removed completely by pipette. The pellet was centrifuged a second time and residual media was aspirated from the pellet. A thin residue was observed on the walls on the micro-centrifuge tube containing the pellet after aspiration so a base-line mass for this residue was obtained by aspirating an equivalent volume of growth media independently. The mass of the resulting pellet was divided by the number of cells in the original volume of media (from standard curve; not shown) to obtain the average cellular mass of *Har. marismortui*. This process was completed three times using 500, 750, and 1250 μL of cell culture. The average cellular volume was then determined using the obtained average cellular mass and cellular density.

### Evaluation of intracellular ion concentrations

Balanced growth cells from 8 mM KCl, 120 mM KCl, and 720 mM KCl media and initial growth cells in alternate ion medias of 120 mM LiCl, 120 mM RbCl, and 120 mM CsCl were pelleted by centrifugation and media removed. Cell pellets were washed twice using the 8 mM KCl media as a means of removing excess ions without lysing cells. Cells were lysed in 5 mL 1 % (v/v) nitric acid and sonicated to break up small particles in the lysate. ICP-MS (University of Northern British Columbia Central Equipment Laboratory, Prince George, British Columbia) was used to determine potassium, lithium, rubidium and cesium concentrations within each cell lysate. The measured concentrations were then used to determine the average moles of ion contributed by a single cell using the straight line equation described above, which in turn was used to determine the intracellular concentration for each ion using the determined cellular volume.

### Genome analysis

A genome search for monovalent cation transporters was performed first by BLAST (Altschul et al. 1990) against the November 2004 release of the *Har. marismortui* genome (Baliga et al. 2004) to identify any of the known potassium transporters. The resultant genes were then self-blasted against the genome for full coverage and identification of any non-annotated genes. Identified genes were then used to determine the gene presence or absence in the selected genome sequences of representative species of other archaeal phyla using the UCSC Archaeal Genome Browser (Chan et al. 2012; Schneider et al. 2006), and the default BLAST search (Kent 2002). The specific genomes used were as follows: March 2010 release of *Hfx*. volcanii (Hartman et al. 2010), October 2000 release of *Halobacterium sp.* NRC-1 (Ng et al. 2000); August 1996 release of *M. jannaschii* (Bult et al. 1996), April 2002 release of *M. acetivorans* (Galagan et al. 2002); December 1997 release of *A. fulgidus* (Klenk et al. 1997), February 2002 release of *P. furiosus* (Robb et al. 2001), October 2001 release of *S. solfataricus* (She et al. 2001), and April 1999 release of *A. pernix* (Kawarabayasi et al. 1999).

## Results

*Haloarcula marismortui* growth was observed across a broad range of extracellular potassium concentrations. To determine cellular generation times the exponential portion of the data was fit to the exponential growth equation *A* = *A*_0_ *e* ^kt^ (Figure A2). Generation times were then plotted against the concentration of potassium in the media to produce a visual representation of growth optima (Fig. 1; Table 1). Generation time assessment confirms that standard growth conditions (120 mM KCl; 23 % S.W. MGM) provided an optimal potassium concentration and produced a generation time of 4.19 ± 0.14 h. The cellular generation time remained highly stable across the elevated potassium concentrations examined; however, as the extracellular potassium concentration decreased the generation time increased sharply.

**Fig. 2:**
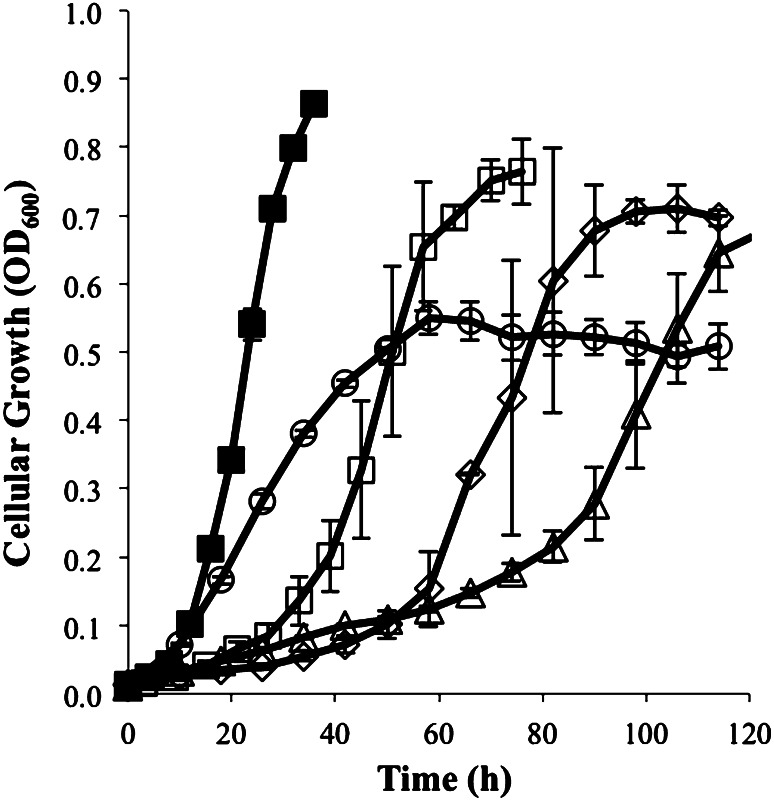
Growth of *Har. marismortui* in alternative ion conditions. Culture growth as measured by optical density in 8 mM KCl (*open square*) 120 mM LiCl (*diamond*), 120 mM RbCl (*circle*), 120 mM CsCl (*triangle*), and 120 mM KCl (*filled square*) media. Alternative ion cultures were inoculated 1:100 with cells grown to balanced growth under standard conditions then incubated at 45 °C until mid-exponential growth was achieved, each is a representative triplicate

Generation times for *Har. marismortui* growth on alternative monovalent ions were determined by inoculating media containing 120 mM concentrations of LiCl, RbCl, or CsCl with mid-exponential, balanced growth *Har. marismortui* cells in standard media as a means of observing growth under sudden stress conditions induced by these ions (Fig. 2; Table 1). When comparing the alternate ion generation times to the 8 mM KCl growth condition, growth in RbCl is similar, while LiCl and CsCl show 2.8- to 3.9-fold increases in the generation times. When investigating the pH profile, cells grew equally at all pH > 5.0, and no growth was observed below pH 5.0 (Figure A1).

The density of *Har. marismortui* cells was determined to be 1.20 g/mL and confirms the result reported by Ginzburg et al. (1970). Additionally, the construction of a standard curve (Figure A3) relating a volume-specific cell count to the optical density has allowed for an approximation of a cellular mass of 1.94 × 10^−12^ ± 0.15 × 10^−12^ g. When used in conjunction with the cellular density, these two values have revealed a cellular volume of 1.62 ± 0.13 fL.

**Fig. 3:**
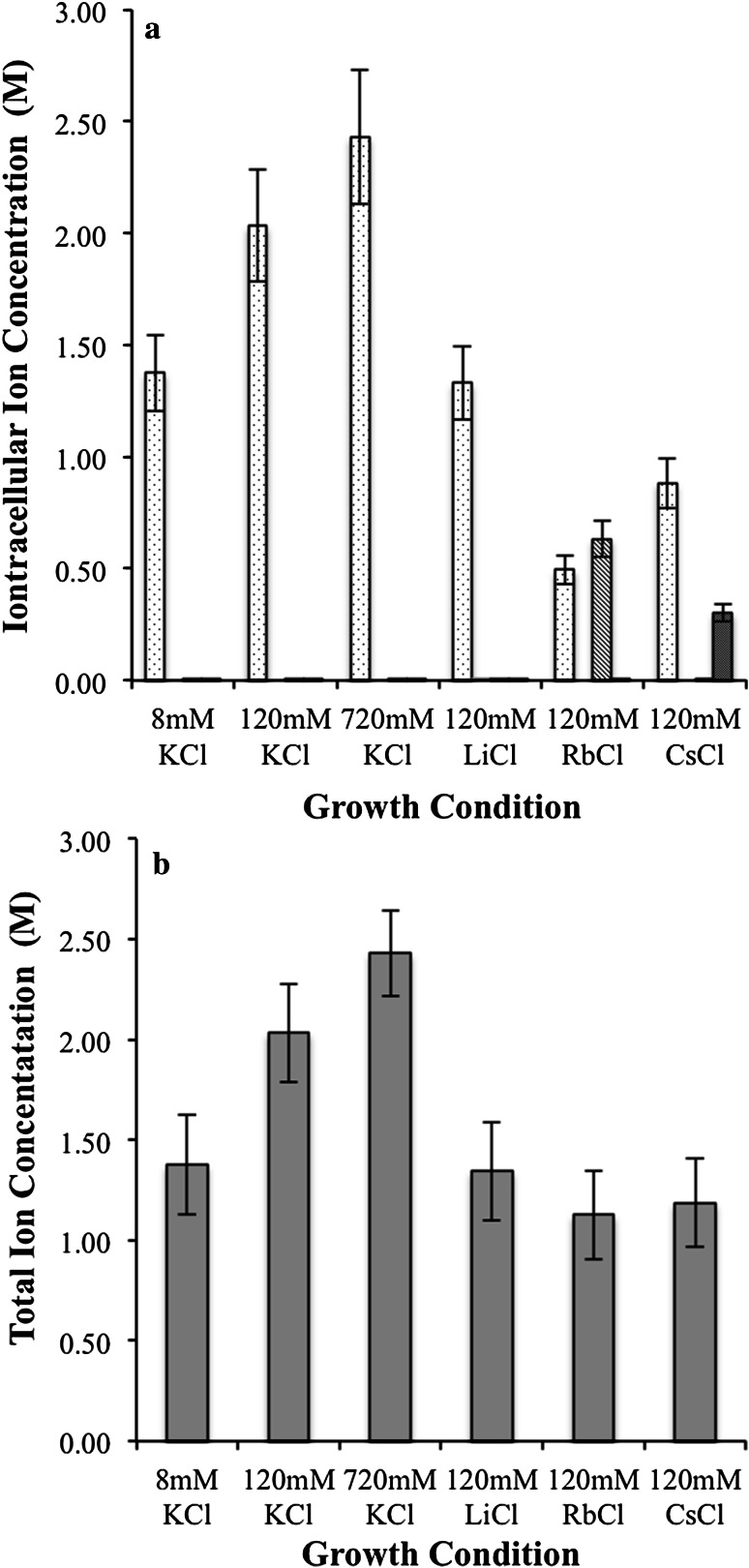
Intracellular ion concentrations. **a** Individual potassium, lithium, rubidium and cesium concentrations, potassium (*light dots*), rubidium (*light gray*), cesium (*dark gray*), and lithium (*below* the scale) concentrations obtained via trace metal analysis with ICP-MS. Concentrations were obtained as described in “Materials and Methods”. **b** Total intracellular ion concentration of monovalent cations. *Har. marismortui* after growth in ion conditions as described. Total ion concentrations are given as the sum of all individual ion concentrations being reported

Intracellular ion concentrations of potassium, lithium, rubidium and cesium were determined by trace metal analysis of cellular lysates using ICP-MS. The ion concentrations within the 5 mL lysates were then used in conjunction with the cell density standard curve and cellular volume to determine the intracellular concentrations for each of the latter ions under various growth conditions (Fig. 3; Table 2). Variation in intracellular concentrations of potassium with respect to changes in extracellular KCl concentration is observed when cells are grown under 120 mM LiCl, RbCl, or CsCl. The individual ion concentrations vary (Fig. 3a) while total ion concentration can be considered as stable, ranging from 1.13 to 1.34 M total ion (Fig. 3b).

**Table 2:** Intracellular monovalent ion concentrations

Growth condition	Ion concentration (mol/L)				
	K^+^	Li^+^	Rb^+^	Cs^+^	Total
8 mM KCl	1.40 ± 0.17	1.40 ± 0.17 × 10^−4^	1.04 ± 0.13 × 10^−4^	1.07 ± 0.13 × 10^−5^	1.38 ± 0.25
120 mM KCl	2.03 ± 0.25	1.10 ± 0.14 × 10^−4^	5.46 ± 0.67 × 10^−5^	1.28 ± 0.16 × 10^−5^	2.03 ± 0.25
720 mM KCl	2.43 ± 0.30	8.37 ± 1.0 × 10^−5^	4.73 ± 0.58 × 10^−5^	ND	2.43 ± 0.21
120 mM LiCl	1.33 ± 0.16	1.44 ± .18 × 10^−2^	9.28 ± 1.1 × 10^−5^	1.25 ± 0.15 × 10^−5^	1.34 ± 0.24
120 mM RbCl	0.49 ± 0.06	ND	0.633 ± 0.081	5.83 ± 0.75 × 10^−6^	1.13 ± 0.22
120 mM CsCl	0.88 ± 0.11	ND	2.45 ± 0.31 × 10^−4^	0.302 ± 0.038	1.19 ± 0.22

Total concentration is given as the sum of all ion concentrations found. Concentrations below the ICP-MS detection limit were given a value of 0.00 when calculating total concentrations

*ND* not detected, below detection limit

Of the known potassium channel families (Choe 2002; Roosild et al. 2004), Trk, Pch (Kef type; voltage gated) and Mth (Kcs type) have been identified in the *Har. marismortui* genome. These channels all fall into the same sub-family of potassium channels. Of note, the Trk system consists of two subunits, minimally (trkA and trkH); but may also contain E and G subunits (Schlosser et al. 1995). In *Har. marismortui*, there are a total of 7 trkA-type genes, and 4 trkH-type genes, while the remainder of the archaeal genomes searched have a subset of these genes and *S. solfataricus* has none. In most genomes, the amino acid transporters are sodium-dependent transporters; however, in the *Har. marismortui* genome all the amino acid transporters are annotated as cation-dependent transporters. Oren (1999) also state that, at the time of his review, a high-affinity potassium transport system similar to the Kdp system (a P-type ATPase potassium pump) found in *E. coli* (Mulder 1986; Oren 1999) had not yet been identified in any of the haloarchaeal species. The genome survey conducted here shows the only haloarchaeal species that possesses a full complement of homologous genes for the Kdp system is *Halobacterium* sp. NRC-1 and interestingly, all of these genes are located on plasmid pNRC200 (Table 3; see Table A1 for complete survey results).

**Table 3:**
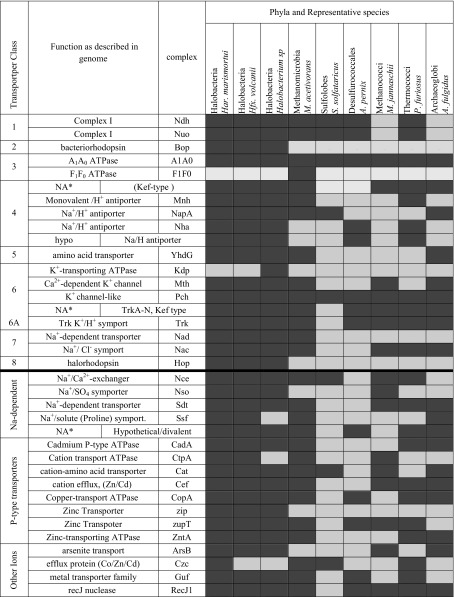
Ion transport systems/complexes identified in archaeal genomes

Dark gray, complex present; light gray complex not identified by genome survey. Phyla designations as from the UCSC Archaeal Genome Browser (Chan et al. 2012; Karolchik et al. 2004; Kent 2002; Schneider et al. 2006). Transporter classes identified as originally assigned by Oren (1999) (above bold line) as follows: 1. Respiratory electron transport, 2. Light driven proton transport, 3. ATP formation, driven by proton gradient, 4. Electrogenic sodium/H+ antiporter, 5. Sodium gradient driven inward aa transport, 6. Potassium uniport (membrane potential driven), 6A. Potassium-Proton Symport, 7. Light independent Cl- transport (likely sodium-coupled), 8. Halorhodopsin (light driven inward Cl-)

*NA not annotated

## Discussion

*Haloarcula marismortui* exhibits an optimal generation time of 4.19 ± 0.14 h under standard conditions (120 mM KCl media; 45 °C, pH 7.5) which agrees with previous generation times of 1.5–3 h in several members of the family *Halobacteriaceae* (Robinson et al. 2005), with some species exhibiting generation times in excess of 6 h (Meury and Kohiyama 1989), indicating this is a typical growth profile. As the potassium concentration in the media was decreased to the minimally attainable 8 mM concentration, the generation time increased to 5.91 ± 0.60 h. The 1.4-fold increase in generation time is likely indicative of an inability to sequester sufficient potassium to maintain proper intracellular functions and is a result of changes to cellular energetics. At the opposite end of the spectrum, as extracellular KCl was increased to 220 mM the generation time increased from optimal to 4.56 ± 0.23 h and slowed only slightly to 4.69 ± 0.33 h at KCl concentrations of 720 mM. The slowed growth at high KCl is occurring due to ionic stress associated with the elevated total ion concentration, sub-optimal function of intracellular systems due to excessive intracellular potassium, or a decrease in the membrane potential. The observed slow growth is likely a consequence of all three.

Cellular volumes of other archaeal species have been reported to range from 0.02 to 0.70 μm^3^ (0.02–0.07 fL) in *Nanoarchaeota equitans* (Baker et al. 2006) to 1700 μm^3^ (1700 fL) in species of *Desulfurococcus* (Steering Group for the Workshop on Size Limits of Very Small Microorganisms 1999). A large number of archaeal phyla have reported cellular volumes in the 1–100 μm^3^ (1–100 fL) range (Steering Group for the Workshop on Size Limits of Very Small Microorganisms 1999) while human erythrocytes have been reported as having a 90 fL volume (McLaren et al. 1987). Even though an attempt to eliminate the mass of the proteinaceous surface layer, proteins, and membranes was not performed in this study as was done by Ginzburg et al. (1970), our obtained volume appears to be a reasonable estimate but is still likely an overestimation. Therefore, the intracellular concentrations determined are likely to better serve as a report of the total concentration of each ion bound by the cell as a whole, and should not be regarded as only the concentration of free intracellular ions.

*Har. marismortui* is tolerant of a 1.38–2.43 M range in intracellular potassium concentrations where the center of this range, 2.03 M, is the optimum (Fig. 3; Table 2). Under minimal potassium conditions (8 mM KCl media), the 1.38 M intracellular potassium demonstrates the ability to scavenge potassium required to osmoregulate from its environment. The increase in generation time for growth at this low extracellular KCl could be indicative of an inability to sequester sufficient potassium to maintain the optimal function of intracellular systems as supported by the decreased intracellular potassium. Potassium efflux has previously been shown to occur through low-to-medium affinity potassium transporters (such as the *E. coli* Trk system) resulting in a futile cycling of potassium ions (Mulder 1986), and a homologous system in *Har. marismortui* indicates additional energy is required to maintain a gradient, suggesting high-affinity, ATP-driven transporters are required. The increased energy requirement to maintain a steep potassium gradient leaves less energy available for other cellular processes and would be exhibited as an increase in generation times, which is what is observed under limiting extracellular potassium concentrations.

**Fig. 4:**
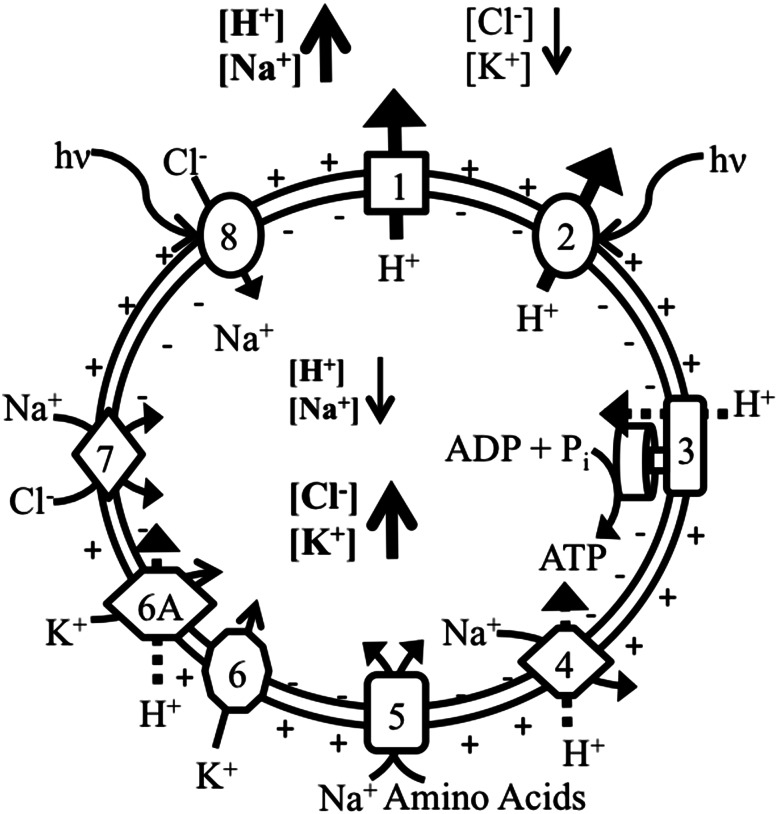
Ion transport systems in* Har. marismortui*. Ion flow in the haloarchaeon, *Har. marismortui* as modeled after Oren et al. (1990) illustrating ion flow within cells. Proton flow in and out of the cell and relative ion concentrations are emphasized to show formation of the proton motive force and ion gradients. Classes are as described in Table 3. The new subclass 6A is defined for K^+^/H^+^ symport, which uses the proton motive force to drive K^+^ sequestration through the Trk system. Sodium transport for class 8 transporters is included as co-transport with chloride (Duschl and Wagner 1986)

The Trk system does not hydrolyze ATP to function, but rather utilizes it for regulatory purposes only (Stewart et al.), and as noted by Oren (1999) it is possible that an increased demand for potassium uptake through the Trk system could immobilize a substantial quantity of ATP. If regulation occurs through the binding of ATP in a transporter active site or allosteric site, the ATP available for other cellular processes would become limited. Additionally, the recognition of Trk as a K^+^/H^+^ symporter (Stewart et al. 1985) allows potassium sequestering to be driven by the proton motive force in *Har. marismortui,* without the requirement for a high-affinity ATPase. This agrees with the finding that no ATP-driven potassium transport was identified in the genome survey and provides a second possible explanation for the observed changes in generation time. Considering the known ion transport systems (Fig. 4), a decrease in total cellular energy availability due to ATP sequestration for the regulation of the Trk symport system would result in a depletion of the cellular ATP pool. This has lead to the sub-division of Oren’s (1999) potassium transport class (class 6; Fig. 4) to a potassium transport and a K^+^/H^+^ symport class (class 6 and 6A, respectively; Fig. 4). It would be worthwhile to investigate changes in the *Har. marismortui* ATP pool through each stage of growth to provide further evidence for this proposed mode of potassium transport and to confirm that these changes in ATP concentration actually occur in response to changes in extracellular potassium concentrations.

From an evolutionary perspective, the Kdp genes found in Halobacterium sp. NRC-1 have likely been obtained from lateral gene transfer from bacterial species as the highest homology occurs in a variety of bacterial species via BLAST with E-scores from 0 to 5e^−106^ (Altschul et al. 1990). The browser also shows the *Halobacterium* sp. NRC-1 *kdpB* gene (VNG6177G) is 30 % homologous to *Har. marismortui* rrnB0270 locus, which codes a zinc-transporting ATPase (Supplementary Table I) and produces a BLAST E-score of 7e^−56^, with similar homology found in *Hfx. volcanii*. Though this E-score is roughly half of those observed in other species with Kdp system genes, and our survey is based solely on existing annotation and homologous similarities, it is possible Kdp homologs exist in *Har. marismortui* but are currently annotated as other ion transporters.

Under high potassium conditions (720 mM KCl) the increase of intracellular potassium (Fig. 3; Table 2) indicates that *Har. marismortui* is unable to regulate the internal salt concentration, sequestering additional ions and producing elevated, sub-optimal internal ion concentrations. Sequestering potassium is expected to require less energy when high potassium is available, so an increase in generation time (a decrease in available cellular energy) due solely to potassium efflux is less plausible, but can be explained when membrane potential (Δ*ψ*_m_) is taken into account. Under these high extracellular potassium concentrations, the Δ*ψ*_m_ is a fraction of the strength of that observed under either standard or limiting potassium conditions. Therefore, the subtle increase in generation time observed can be attributed to lower energy generation from this limited Δ*ψ*_m_. A second explanation is that increased overall ionic stress, as extracellular NaCl was not decreased when KCl was increased, further decreases the Δ*ψ*_m_, and potassium accumulation through ion-channels is a consequence of the membrane potential and sequestration of chloride.

A collapse of the membrane potential can also explain the lack of observable growth under low-pH conditions as several Na^+^/H^+^ antiporters are pH-regulated (Kosono et al. 2005; Padan et al. 2004) and become inactive below a pH of 6–7 in other species. The lack of growth below a pH of 5 for *Har. marismortui* could be a result of the inability to regulate sodium transport, resulting in a collapse of the membrane potential. Signaling pathways affected by ionic stress in both bacterial and eucaryal cells have been widely reported, and may have a profound effect on generation time.

To investigate if the growth changes are due to an ion specificity, alternative monovalent ion growth was determined. Rubidium has been previously used as an ion transport study tool as *Hfx. volcanii* was shown to uptake rubidium during potassium starvation (Meury and Kohiyama 1989). This attribute was exploited in an attempt to estimate the quantity of permanently bound potassium within these cells by forcing a maximum quantity of mobile potassium ions out of the cell via exchange with rubidium (Meury and Kohiyama 1989). The authors suggested that 50 % of the potassium in *Hfx. volcanii* is exchangeable with rubidium which indicates that up to 1.8 M potassium is free and non-bound while the remaining potassium (up to 1.5 M) is tightly bound within cells (Meury and Kohiyama 1989). Our findings agree, as the intracellular potassium concentration dropped to 0.494 ± 0.06 M while intracellular rubidium increased to 0.633 ± 0.081 M when cells were grown in 120 mM RbCl media. A similar result is observed when cells are grown in the presence of cesium, albeit to a lesser extent. Cesium is sequestered internally to concentrations of 0.302 ± 0.038 M while the potassium concentration falls to 0.883 ± 0.112 when *Har. marismortui* is grown in the presence of 120 mM CsCl (Fig. 3a). This is again quite likely an unbiased accumulation of both ions as a means of maintaining osmoregulation.

The similar chemistries and potentially similar hydration of these ions may allow for transport of cesium into the cell via established potassium transporters. Previous reports have shown that specific hydration patterns are required within the transport channel in order for potassium transport channels to accurately select potassium ions as they are moved across the cell membrane in *E. coli* (Zhou et al. 2001). The ATP-regulated K^+^/H^+^ symporter identified as a Trk system may provide additional merit to this argument, as previous work with the *E. coli* Trk system, specifically the low-affinity potassium uptake protein *trkd* is capable of transporting rubidium and cesium in addition to potassium in some bacterial species, but only potassium or rubidium in others (Bossemeyer et al. 1989; Brown and Cummings 2001). The presence of this system in members of the family *Halobacteriaceae* makes the transport of the alternative ions via this transport channel quite plausible.

The high chemical and size similarity of rubidium to potassium explains the similar intracellular concentrations observed of these two ions when grown in 120 mM RbCl, while the substantially larger size of the cesium ion makes transport of this ion more difficult, and thus a lower intracellular cesium concentration is observed when 120 mM CsCl media is used. As the lowest attainable potassium concentration remains conservatively estimated at 8 mM, it is still possible that the apparent exchange of rubidium or cesium for mobile potassium ions is actually a general accumulation of all these ions from the media. *Hfx. volcanii* and *Har. marismortui* are likely accumulating both potassium and either rubidium or cesium simultaneously without or with minimal bias as a means of osmoregulation thus reiterating the need for analysis of contaminants in studies of this nature.

The accumulation of lithium as an alternative to potassium does not appear to occur when grown in 120 mM LiCl; however, lithium concentrations (0.0144 ± 0.0018 M, Fig. 3a) may not be accurate due to the space-charge effects that can be observed when conducting ICP-MS analysis of lighter ions (Maher et al. 2001; Olesik and Dziewatkoski 1996). These effects occur with the mutual repulsion of positively charged ions, which forces ions out of the argon plasma laterally within the instrument, resulting in an overall lower sensitivity and occurs more noticeably at high ion concentrations making ICP-MS analysis of lithium difficult and less reliable. While this could imply the actual intracellular concentration of lithium is higher, there is no observed decrease in potassium concentration compared to those grown solely under 8 mM KCl, as was observed with growth in the presence of RbCl and CsCl (Fig. 3), suggesting it is unlikely that *Har. marismortui* is sequestering lithium under potassium-limiting conditions. It is more plausible that cells are scavenging potassium from the extracellular media when grown in the presence of lithium and the ion is excluded from the cell due to the smaller size of the ion.

The resilience and adaptability of *Har. marismortui* to respond to changes in its native environment are clearly demonstrated through the cellular generation times under high and low potassium stress, and these are reflected in changes to intracellular ion concentrations. In minimal potassium media, intracellular potassium is reduced by 30 %, presumably near the lower concentration limit for cellular functions, and the drastically increased generation times observed can be attributed to a decrease in available energy due to the amount of additional ATP immobilized by the Trk system for regulation. In high potassium media, intracellular potassium increases by 25 %, generation times increase consistently but are sub-optimal, and total salt saturation is reached. We suggest that potassium uptake is driven by a proton motive force through a Trk K^+^/H^+^ symport system, and the observed generation times can be explained through the loss of the membrane potential.

## Electronic supplementary material

Below is the link to the electronic supplementary material.

Supplementary material 1 (PDF 3319 kb)

Supplementary material 2 (PDF 139 kb)

## Abbreviations

ICP-MS: Inductively coupled plasmas mass spectrometry
OD: Optical density
S.W. MGM: 23 % Salt water modified growth media
