# Supplementary material for: Potassium stress growth characteristics and energetics in the haloarchaeon Haloarcula marismortui
Source: Extremophiles. 2014 Dec 11;19(2):315–25. doi: 10.1007/s00792-014-0716-z (PMC4339784; doi:10.1007/s00792-014-0716-z)
Supplement: Supplementary file 1 — Supplementary material 1 (PDF 3319 kb) [file 792_2014_716_MOESM1_ESM.pdf]

Supplemental Table I. Genome survey for potassium transporters. Numbered transporter classes are as assigned by Oren (Oren 1999) and are as follows: 1.Respiratory electron transport 2.Light driven proton transport 3.ATP formation, driven by proton gradient 4.Electrogenic Na^+/H^+ antiporter 5.Sodium-gradient driven inward amino acid transport 6.Potassium uniport (membrane potential driven) 6A.Potassium-Proton Symport 7.Light-independent Cl^- transport (likely Na^+ coupled) 8.Halorhodopsin (light driven inward Cl^-). Additional transporters are described in column. Genome search performed as described in Materials and Methods, comparing to the identified *Har. marismortui* protein sequence, with only the highest scoring hit given for the other organisms if more than one hit was found.

**Transporter Class**	**Description of Membrane-bound Protein or Complex**	**Gene Name**	**Phyla and Representative species**								
			Halobacteria *Haloarcula marismortui*	Halobacteria *Haloferax volcanii*	Halobacteria *Halobacterium sp*	Methanomicrobia *Methanosarcina acetivorans*	Sulfolobes *Sulfolobus solfataricus*	Desulfurococcales *Aeropyrum pernix*	Methanococci *Methanocaldococcus jannaschii*	Thermococci *Pyrococcus furiosus*	Archaeoglobi *Archaeoglobus fulgidus*
	NADH dehydrogenase subunit 4	ndhD	rrnAC1456	HVO_0987	VNG0647G	MA1505	-	APE_1415	-	-	-
	monovalent cation/H+ antiporter subunit D/ chain M (nadh:ubi ox subnit4)	ndhG2	rrnAC3531	HVO_1066	VNG0563G	MA1506	-	-	MJ1309	PF1447	-
1	NADH dehydrogenase/oxidoreductase	ndhG3	rrnAC1458	HVO_0988	VNG0648G	MA1506	SSO0329	-	-	PF1446	-
	NADH dehydrogenase subunit J	ndhG6	rrnAC1452	HVO_0983	VNG0641C	MA1501	-	-	-	-	-
	resistance to high Na+, K+, Li+ and/or alkali; transfer of e- from NADH to ubiquinone (ShaA?)	nuoL1	rrnAC3530	HVO_1069	VNG0562C	MA4572	-	-	-	PF1430	-

	NADH dehdrogenase subunit L (complex I; 1450-1455region)	nuoL2	rrnAC1455	HVO_0986	VNG0646G	MA1504	-	APE_1411.1	-	-	-
2	bacteriorhodopsin	bop	rrnAC3161	-	VNG1467G	-	-	-	-	-	-
3	ATPsynthase Subunit C (A1A0)	aptC2	rrnac1435	HVO_0312	VNG2143G	MA4154	SSO0567	APE_2326.1	MJ0221	PF0178	AF1160
	H+ ATP synthase subunit C (A1A0)	atpC1	rrnac3155	HVO_0312	VNG2143G	MA4154	SSO0567	APE_2326.1	MJ0221	PF0178	AF1160
	V-type ATP synthase subunit D (A1A0)	atpD	rrnac3162	HVO_0319	VNG2135G	MA4160	SSO0566	APE_0402.1	MJ0615	PF0184	AF1168
	V-type ATP synthase subunit E (A1A0)	atpE	rrnac3156	HVO_0313	VNG2142G	MA4155	-	-	MJ0220	PF0179	AF1163
	V-type ATP synthase subunit F (A1A0)	atpF	rrnac3158	HVO_0315	VNG2140G	MA4157	-	-	-	PF0181	AF1165
	vacuolar (H+)-ATPase G subunit (A1A0)	atpG	rrnac3152	HVO_0310	VNG2146H	MA4152	-	APE_1708	MJ0223	PF0176	AF1158
	V-type ATP synthase subunit I (A1A0)	atpI	rrnac3154	HVO_0311	VNG2144G	MA4153	SSO0559	APE_0673.1	MJ0222	PF0177	AF1159
	V-type ATP synthase subunit A (A1A0)	ntpA	rrnac3159	HVO_0316	VNG2139G	MA4158	SSO0563	APE_0405.1	MJ0217	PF0182	AF1166
	V-type ATP synthase subunit B (A1A0)	ntpB	rrnac3160	HVO_0317	VNG2138G	MA4159	SSO0564	APE_0404.1	MJ0216	PF0183	AF1167
	V-type ATP synthase subunit C (A1A0)	ntpC	rrnac3157	HVO_0314	VNG2141G	MA4156	-	-	MJ0219	PF0180	AF1164
	H(+)-transporting ATP synthase, subunit gamma	F1F0	-	-	-	MA2433	-	-	-	-	-
	F0F1 ATP synthase subunit alpha	F1F0	-	-	-	MA2434	-	-	-	-	-
	H(+)-transporting ATP synthase, subunit B	F1F0	-	-	-	MA2435	-	-	-	-	-
	F0F1 ATP synthase subunit C	F1F0	-	-	-	MA2436	-	-	-	-	-
	F0F1 ATP synthase subunit A	F1F0	-	-	-	MA2437	-	-	-	-	-
	F0F1 ATP synthase subunit epsilon	F1F0	-	-	-	MA2440	-	-	-	-	-

	F0F1 ATP synthase subunit beta	F1F0	-	-	-	MA2441	-	-	-	-	-
	Not Annotated, Kef-type transporter		rrnAC1272	HVO_0990	VNG0650C	MA1171	-	APE_1437.1	MJ0608	PF1942	AF0756
	putative monovalent cation/H+ antiporter subunit tD / NADH dehydrogenase (quinone)	Mnh	rrnAC3529	HVO_1069	VNG0560C	MA4368	-	-	-	PF1430	-
	putative monovalent cation/H+ antiporter subunit E	mnhE	rrnAC3537	HVO_1065	VNG0571C	MA4568	-	-	-	PF1453	-
	putative monovalent cation/H+ antiporter subunit B	mnhG1	rrnAC3533	HVO_1068	VNG0564H	MA4665	-	-	-	PF1149	-
	putative monovalent cation/H+ antiporter subunit G (MnhG subunit)	mnhG2	rrnAC3535	HVO_1063	VNG0570Hm	MA4566	-	-	-	PF1451	-
	putative monovalent cation/H+ antiporter subunitF	MrpF	rrnAC3536	HVO_1064	VNG0568C	-	-	-	-	PF1152	-
	Na(+)/H(+) antiporter	napA	rrnAC3025	HVO_0051	-	MA3981	SSO1138	-	-	-	AF1245
4	Na(+)/H(+) antiporter	nhaC1	rrnAC0229	HVO_1394	VNG0436G	-	-	-	-	PF2032	-
	Na+/H+ antiporter	nhaC2	rrnAC0143	HVO_2093	VNG6313G	-	-	APE_2538	-	PF2032	-
	Na/hydrogen antiporter	nhaC3	rrnAC1870	HVO_1262	VNG2123G	-	-	APE_2538	-	PF2032	-
	Na/hydrogen antiporter	nhaC4	pNG7169	HVO_2093	VNG2123G	-	-	APE_2538	-	PF2032	-
	Na/hydrogen antiporter	nhaC5	rrnAC0180	HVO_1605	VNG2124C	-	-	APE_2538	-	PF2032	-
	Na+/H+ antiporter	nhaC6	rrnAC2558	HVO_1916	VNG2068C	-	-	-	MJ1105	-	AF1673
	Not annotated, Na/hydrogen antiporter		rrnAC3446	HVO_1605	VNG2124C	-	-	APE_2538	-	PF2032	-
	putative monovalent cation/H+ antiporter subunit C	mnhC	rrnAC3532	HVO_1067	VNG0565C	MA4570	-	-	MJ1310	PF1448	-
	putative monovalent cation/H+ antiporter subunit B	mnhB	rrnAC3534	HVO_1069	VNG0566C	MA4572	-	-	MJ0437	PF1450	-
5	amino acid transporter	yhdG	rrnAC1651	HVO_0176	VNG1240G	MA3379	-	-	-	-	AF1612

6	potassium-transporting ATPase subunit A	kdpA	-	-	VNG6176G	-	-	-	-	-	-
	potassium-transporting ATPase subunit B	kdpB	-	HVO_1751	VNG6177G	MA3632	-	-	-	PF0740	AF0152
	potassium-transporting ATPase subunit C	kpbC	-	-	VNG6178G	-	-	-	-	-	-
	Ca2+-activated channel mthK	mthK	rrnAC2811	HVO_1916	VNG2068C	MA3020	-	-	MJ1105	-	AF0838
	Ca2+-activated channel mthK2	mthK2	rrnAC2606	HVO_2616	VNG1924G	-	-	-	MJ0138.1	-	AF1673
	potassium channel-like (Kef)	pch1	pNG7323	HVO_2616	VNG1847G	MA2447	SSO1757	-	MJ0138.1	PF0175	AF1673
	potassium channel-like	pch2	rrnAC3436	HVO_2616	VNG1847G	MA2448	SSO1757	APE_0955	MJ1357	-	-
	potassium channel-related protein	pchB	rrnAC2512	HVO_0467	VNG2104G	MA1435	-	APE_1999	MJ1357	PF0146	AF2197
6A	Trk potassium uptake system protein	trkA	rrnAC1536	HVO_1058	VNG6175G	MA2210	-	APE_2456.1	MJ1105	PF0175	AF0838
	Trk potassium uptake system protein	trkA1	rrnAC0754	HVO_1055	VNG0175G	MA2210	-	APE_2456.1	MJ1105	PF0175	AF0838
	Trk potassium uptake system protein	trkA3	rrnAC3289	HVO_2211	VNG1924G	MA1482	-	-	MJ1105	PF0175	AF0838
	Trk potassium uptake system protein	trkA4	rrnAC0245	HVO_2211	VNG6218G	MJ1105	-	APE_2456.1	MJ1105	PF0175	AF0838
	Trk potassium uptake system protein	trkA5	rrnAC1652	HVO_1916	VNG2068C	-	-	-	-	-	-
	Trk potassium uptake system protein	trkA6	rrnAC2498	HVO_1885	VNG1924G	MA2210	-	-	MJ1105	PF0175	-
	Trk potassium uptake system protein	trkA7	rrnAC0243	HVO_2211	VNG6223C	MA2210	-	APE_2456.1	MJ0138.1	PF0175	AF0838
	rrnAC1107	trkH1	rrnAC1107	HVO_1057	VNG2565G	MA1483	-	APE_0715.1	MJ1485	PF1856	AF0839
	trkH2	trkH2	rrnAC0757	HVO_1057	VNG1284G	MA1483	-	APE_0715.1	MJ1485	PF1856	AF0839
	trkH2	trkH2	rrnAC2510	HVO_1137	VNG0615C	MA2448	-	-	-	-	AF1673

	trkH3	trkH3	rrnAC0755	HVO_1056	VNG1721G	MA2209	-	APE_0715.1	MJ1485	PF1856	AF0839
	Not annotated<br>TrkA-N/Kef type transporter		rrnAC0744	HVO_1690	VNG0983C	MA1171	-	APE_1437.1	MJ0988	PF1942	AF2029
7	Not annotated<br>sodium symporter (nadC family)		rrnAC2632	HVO_A009<br>1	-	-	-	-	-	-	-
	sodium-and chloride-dependent transporter:symport (Sodium:neurotransmitter symporter family)	nac	rrnAC0010	HVO_2469	VNG1526G	MA0901	-	-	MJ1319	PF1254	AF1995
	sodium dependent transporter	nadC2	rrnB0036	HVO_1920	VNG2602G	-	-	-	MJ0672	-	-
8	halorhodopsin	hop	rrnAC1659	-	VNG0180G	-	-	-	-	-	-
Na-dependent Transporters	Na+Ca+ exchanging protein	nce2	rrnAC0986	HVO_2355	VNG2257G	MA2008	-	-	MJ0091	PF0350	-
	Na+/Ca2+-exchanging protein (cation/H+ antiporter)	nce3	rrnAC3276	HVO_2155	VNG2257G	MA2008	SSO1174	-	MJ0091	PF0350	-
	sodium/sulfate symporter family transporter	nso	rrnAC2759	HVO_0282	VNG2602G	-	-	-	MJ0672	-	-
	sodium dependent transporter	sdt	pNG7117	HVO_2470	VNG6261G	MA0901	-	-	MJ1319	PF1254	AF1995
	sodium/solute symporter<br>links to actP/also noted as a Na/proline	ssf	rrnAC3226	HVO_A015<br>4	-	MA1316	-	-	-	-	AF0969
	Not annotated<br>Divalent transporter		rrnAC3134	HVO_0124	VNG2458C	MA1437	-	APE_0156.1	-	PF1157	AF1505
P-type transporters	Cadmium transporting P-type ATPase	cadA	pNG6019	HVO_A062<br>4	VNG0149G	MA3632	-	-	-	PF0740	AF0152
	cationic amino acid transporter	cat	rrnAC0759	HVO_0176	VNG0174G	MA2286	SSO1463	-	MJ0609	-	AF1612
	cation efflux protein (Co/Zn/Cd)	cat	rrnAC2721	HVO_0244	-	MA3366	-	-	MJ0449	PF0558	AF2388
	divalent cation transporter	cat	rrnAC3135	HVO_0125	VNG2456C<br>m	-	-	APE_0156.1	-	PF1157	AF1505
	cationic amino acid transporter<br>NaKClcotrasnporter	cat1	pNG7063	HVO_2500	VNG0174G	MA3379	SSO1463	-	-	-	AF1612

	cationic amino acid transporter	cat3	rrnAC0244	HVO_A0175	VNG6220G	MA2286	SSO1463	APE_0010.1	MJ0609	-	AF1612
	cation efflux system (Zn/Cd)	cef	rrnAC3425	HVO_2602	VNG1862G	MA0549	-	-	MJ0449	PF1373	AF2137
	cation-transporting ATPase, ATPase, P-type (transporting), HAD superfamily, subfamily IC	ctpA	pNG7137	HVO_0933	-	MA4082	-	-	MJ1226	-	-
	cation transporting ATPase	ctpB	rrnAC1149	HVO_0940	VNG2201G	MA1342	-	-	-	-	AF0473
	cation-transporting ATPase	ctpC	pNG6135	HVO_A0624	VNG0149G	MA3632	-	-	-	PF0740	AF0473
	Copper transporting ATPase	copA1	pNG6059	HVO_0940	VNG2201G	MA1342	-	APE_1454.1	-	PF0740	AF0152
	Copper transporting ATPase	copA2	pNG6056	HVO_1751	-	MA0166	-	-	-	PF0740	AF0473
	Copper transporting ATPase	copA3	pNG6046	HVO_1751	VNG0700G	MA1342	-	APE_1454.1	-	PF0740	AF0473
	Copper transporting ATPase	copA4	rrnAC0504	HVO_1751	VNG0700G	MA1342	-	APE_1454.1	-	PF0740	AF0473
	Copper transporting ATPase	copA5	pNG6161	HVO_1751	VNG0700G	MA1342	-	-	-	PF0740	-
	Zinc Transporter	zip	rrnAC0129	HVO_2531	VNG1744H	-	-	-	-	-	-
	Zinc Transpoter	zupT	rrnAC2244	HVO_0005	VNG0938G	MA0387	-	APE_1691	-	PF0746	-
	Zinc-transporting ATPase	zntA1	pNG6081	HVO_A0624	VNG0149G	MA3632	-	-	-	PF0740	AF0473
	Zinc-transporting ATPase	zntA2	rrnB0270	HVO_A0624	VNG0149G	MA3632	-	-	-	PF0740	AF0473
	Zinc transporting ATPase	zntA3	rrnAC0298	HVO_A0624	VNG0149G	MA3632	-	-	-	PF0740	AF0473
Other Cation Transport	arsenite transport protein	arsB	rrnAC3466	HVO_1998	VNG2602G	-	-	-	MJ0672	-	AF2308
	cation efflux system protein (Co/Zn/Cd)	czcD	pNG6079	-	-	MA0805	SSO2247	-	-	PF0558	-
	metal transporter family GufA	gufA	rrnAC3110	HVO_1003	VNG0938G	MA2881	-	APE_1691	-	PF0746	-

	recJ nuclease (Trk-N domain containing)	recJ1	rrnAC1311	HVO_0990	VNG6183C	MA1171	-	APE_1437.1	MJ0988	PF1942	AF2029
