# Supplementary material for: Potassium stress growth characteristics and energetics in the haloarchaeon Haloarcula marismortui
Source: Extremophiles. 2014 Dec 11;19(2):315–25. doi: 10.1007/s00792-014-0716-z (PMC4339784; doi:10.1007/s00792-014-0716-z)
Supplement: Supplementary file 2 — Supplementary material 2 (PDF 139 kb) [file 792_2014_716_MOESM2_ESM.pdf]

Supplemental Figures for:

Potassium Stress Growth Characteristics and Energetics in the Haloarchaeon *Haloarcula marismortui*

Journal: Extremophiles

Matthew W. Jensen[1], Scott A. Matlock[1], Carlene H. Reinheimer[1], Caleb J. Lawlor[1], Travis A. Reinheimer[1] and Andrea Gorrell[1*]

[1]Department of Chemistry, University of Northern British Columbia, 3333 University Way, Prince George, BC, V2N 4Z9.

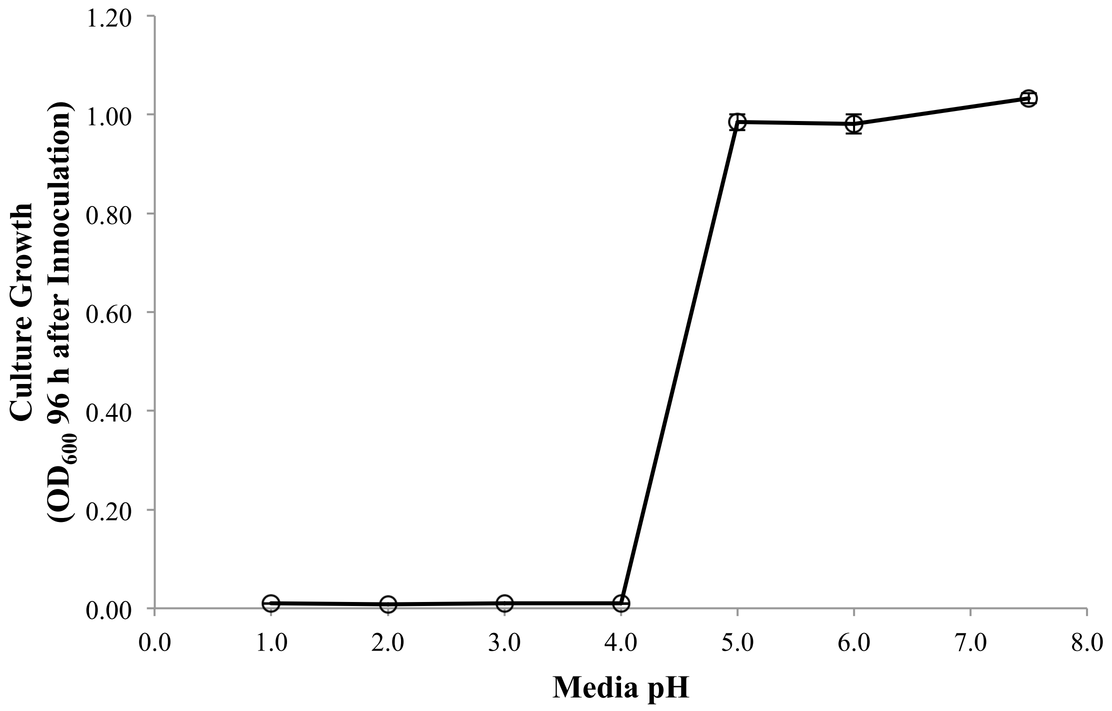

Supplemental Figure A1. **Growth profile of *Har. marisnortui* under varying pH conditions**. Cultures inoculated 1:100 with cells in balanced growth under standard conditions (pH 7.5) and measured after 96h growth in standard media with pH as indicated in triplicate. Media pH was adjusted using HCl.

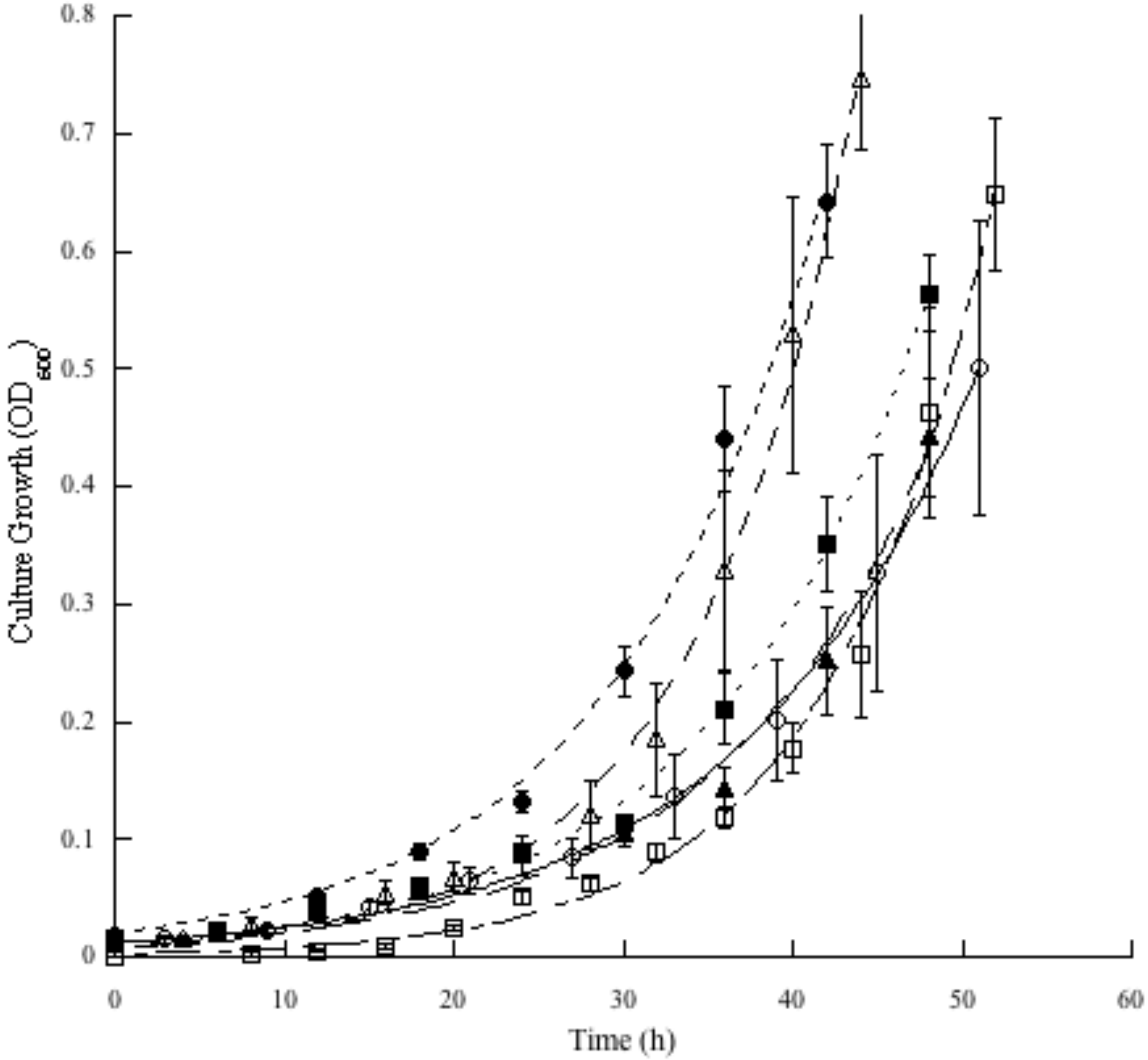

Supplemental Figure A2. Example fit of growth curve. Generation time calculated for each concentration from fit of $A = A_0 e^{kt}$ where A= measured OD_{600}; A_0 = initial OD_{600}, k= growth constant; t= generation time. Cultures were inoculating 1:100 with cells in balanced growth under standard media conditions. 8 mM KCl (open circle), 30 mM KCl (open square), 120 mM (open triangle), 320 mM KCl (filled circle), 520 mM KCl (filled square), 720 mM KCl (filled triangle).

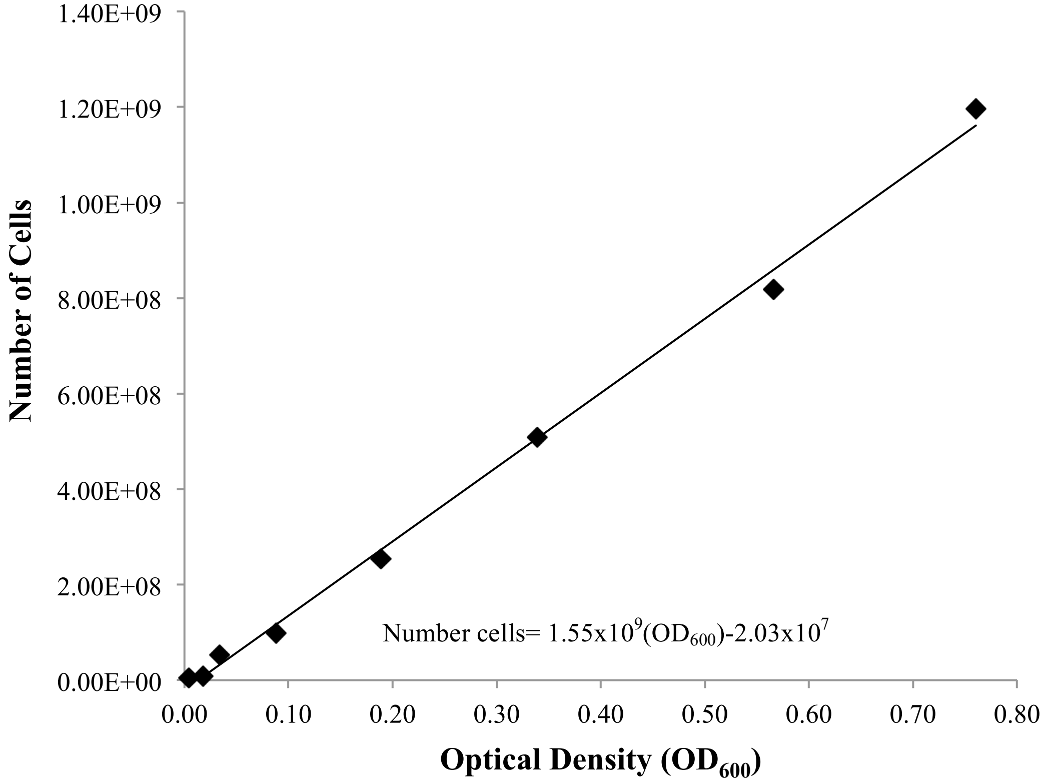

Supplemental Figure A3. **Cell Density Standard Curve**. Linear regression fit to the data is used for calculation of individual cell mass and individual cell volume. Cells counts were taken from cells in balanced growth with standard media.
